# Synthesis of Lipophilic Esters of Tyrosol, Homovanillyl Alcohol and Hydroxytyrosol

**DOI:** 10.3390/antiox8060174

**Published:** 2019-06-14

**Authors:** Roberta Bernini, Isabella Carastro, Francesca Santoni, Mariangela Clemente

**Affiliations:** Department of Agriculture and Forest Sciences (DAFNE), University of Tuscia, Via S. Camillo de Lellis, 01100 Viterbo, Italy; isabella109@alice.it (I.C.); santonifrancesca@yahoo.it (F.S.); marian.clem@unitus.it (M.C.)

**Keywords:** tyrosol, homovanillyl alcohol, hydroxytyrosol, dimethyl carbonate, lipophilic alkyl esters, hydroxytyrosol-enriched extracts, *Olea europaea*, green chemistry, circular economy

## Abstract

Low-molecular weight phenols such as tyrosol, homovanillyl alcohol and hydroxytyrosol are valuable compounds that exhibit a high number of health-promoting effects such as antioxidant, anti-inflammatory and anticancer activity. Despite these remarkable properties, their applications such as dietary supplements and stabilizers of foods and cosmetics in non-aqueous media are limited for the hydrophilic character. With the aim to overcome this limitation, the paper describes a simple and low-cost procedure for the synthesis of lipophilic esters of tyrosol, homovanillyl alcohol and hydroxytyrosol. The reactions were carried out under mild and green chemistry conditions, at room temperature, solubilizing the phenolic compounds in dimethyl carbonate, an eco-friendly solvent, and adding a little excess of the appropriate C2–C18 acyl chloride. The final products were isolated in good yields. Finally, according to the “circular economy” strategy, the procedure was applied to hydroxytyrosol-enriched extracts obtained by *Olea europaea* by-products to prepare a panel of lipophilic extracts that are useful for applications where solubility in lipid media is required.

## 1. Introduction

**Figure 1:**
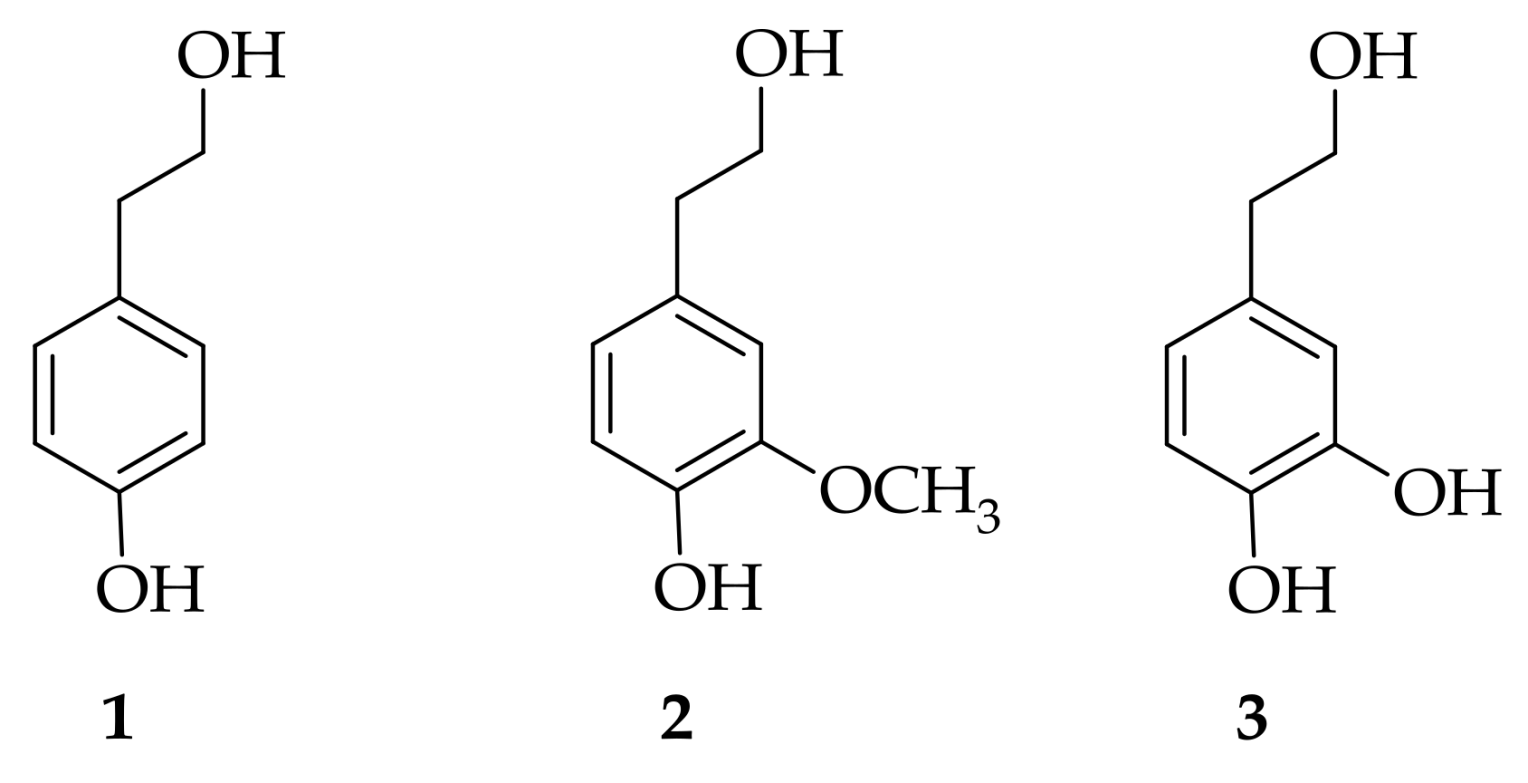
Low-molecular weight phenols found in olive mill wastewater.

Tyrosol **1** (4-hydroxyphenethyl alcohol), homovanillyl alcohol **2** (3-hydroxy-4-methoxyphenethyl alcohol) and hydroxytyrosol **3** (3,4-dihydroxyphenethyl alcohol) are low-molecular weight phenols (Figure 1) found mainly in olive mill wastewater, the by-products of the olive oil production [1,2], from which they can be extracted using environmentally and economically sustainable technologies [3,4] and reused according to the circular economy strategy [5].

They are valuable compounds, displaying a high number of health-promoting effects such as antioxidant, anti-inflammatory and anticancer activity [6,7]. In particular, tyrosol **1** suppresses allergic inflammatory disorders [8], and prevents apoptosis in irradiated keratinocytes [9]. In addition, it is useful to synthetize novel antioxidants [10,11]. Homovanillyl alcohol **2** is a good antioxidant agent [12] and exhibits anti-inflammatory effects on human gastric adenocarcinoma cells [13]. In accordance with the theoretical predictions on *ortho*-diphenols [14], hydroxytyrosol **3** is the most powerful antioxidant found in olive oil by-products. In addition, it increases high-density lipoprotein (HDL)-cholesterol, inhibits inflammation, improves endothelial function, decreases tumor growth and metastasis, and protects the central nervous system [15,16,17,18]. In our experiment, hydroxytyrosol was synthetized by the 2-iodoxybenzoic acid (IBX) oxidation of tyrosol by safe and eco-friendly procedures [19,20] and used to prepare both hydroxytyrosol-derived compounds [21,22,23] and poly(vinyl alcohol)-based films with antioxidant activity [24,25,26].

Despite the remarkable biological properties of these compounds, their applicability as active ingredients in lipophilic foods and cosmetic products requiring solubility in non-aqueous media is limited for the hydrophilic character. With the aim to overcome this limitation, common for almost all phenolic compounds, recently, a growing interest has been devoted to synthesizing lipophilic derivatives. They could be prepared incorporating one or more halogen atoms [27,28,29] or by introducing different chain lengths [30,31,32,33,34,35,36,37,38,39,40] into the molecular skeleton, avoiding the derivatization of the phenolic moiety to which the biological effects, and in particular the antioxidant activity, are attributed.

Among them, tyrosyl, homovanillyl and hydroxytyrosyl esters with different chain lengths proved promising for their beneficial effects in non-aqueous media related to their lipophilicity and bioavailability. In particular, antioxidant activity and this relationship with chain length were the objects of many studies. In emulsified systems, the antioxidant activity of alkyl esters is directly related to the chain length: it increases up to a point (C8–C10) and then dramatically decreases [33], revisiting the polar paradox theory [41]. This effect, named “the cut-off effect”, was explained with the surfactant property of lipophilic derivatives. C8–C10 hydroxytyrosyl esters are both the best antioxidants and surfactants [33]. Other studies have shown that esters with medium length chains have comparable or higher antioxidant activity than esters with long chains [42]. Tyrosyl esters were tested as antimicrobial and antileishmanial agents [35] and, recently, their absorption and stability in the intestinal lumen were investigated [43]. This study has demonstrated that lipophilic tyrosol esters were well absorbed by the intestinal lumen, confirming the relevant role of the alkyl chain [43]. Lipophilic hydroxytyrosol esters are good candidates as active ingredients of formulations for the treatment of cutaneous inflammations, permeating through the human stratum corneum and viable epidermis membrane and releasing hydroxytyrosol, which is the antioxidant and anti-inflammatory agent [44].

Based on the literature data, and the need to prepare lipophilic phenolic derivatives for extending their use in food and cosmetic sectors, we planned a simple and low-cost procedure to obtain tyrosyl, homovanillyl and hydroxytyrosyl alkyl esters. Finally, the procedure was applied to hydroxytyrosol-enriched extracts obtained by *Olea europaea* by-products to prepare a panel of lipophilic extracts that are useful for applications where solubility in lipid media is required.

## 2. Materials and Methods

### 2.1. Chemicals

Solvents, reagents, standards, silica gel 60 F254 plates, and silica gel 60 were purchased from Sigma-Aldrich (Milan, Italy). Hydroxytyrosol was prepared by the IBX oxidation of tyrosol as already reported [19]. A patented procedure based on membrane technology was applied to obtain hydroxytyrosol-enriched extracts from *Olea europaea* by-products [45]. The vegetal material was extracted with water and then subject to microfiltration (MF), nanofiltration (NF), and reverse osmosis (RO). Microfiltration was carried out with tubular ceramic membranes in titanium oxide; nanofiltration and reverse osmosis were carried out using spiral wound module membranes of poly(ether sulfone) [45]. The collected fraction was concentrated using a heat pump evaporator (Vacuum Evaporators—Scraper Series, C&G Depurazione Industriale Srl, Firenze, Italy) [3,4]. High Performance Liquid Chromatographic (HPLC) analysis of the resulting extracts was carried out at λ = 280 nm using a diode array detector (DAD) to identify the polyphenolic compounds found in the sample. The quantitative data were referred to pure standards (tyrosol, hydroxytyrosol, oleuropein, and caffeic acid).

### 2.2. Instruments

NMR spectra (^1^H NMR, ^13^C NMR) were recorded using a 400-MHz nuclear resonance spectrometer Advance-III Bruker (Munich, Germany). Each sample (20 mg) was dissolved in chloroform-d3 or methanol-d4 (0.5 mL). The chemical shifts were expressed in parts per million (δ scale) and referenced to either the residual protons or carbon of the solvent.

HPLC analysis of the hydroxytyrosol-enriched extracts and the corresponding lipophilic fractions were carried out using a HP 1200 liquid chromatograph (Agilent Technologies, Palo Alto, CA, USA). The detector was a diode array and the column was a LiChrosorb RP-18 (250 × 4.60 mm, 5 μm i.d.; Merck, Darmstadt, Germany). A flow rate of 0.8 mL min^−1^ was used for 88 min working from 100% of solvent A (H_2_O at pH = 3.2) to 100% of solvent B (CH_3_CN).

### 2.3. Synthesis of Esters: General Procedure

The appropriate phenethyl alcohol (0.5 mmol) was solubilized in dimethyl carbonate (1.5 mL) at 25 °C. Then the acyl chloride was added (0.6 mmol) and the mixture was kept under stirring for 24 h. The reaction was monitored by thin-layer chromatography on silica gel plates using mixtures of dichloromethane and methanol (9.8/0.2, 9.5/0.5 or 9.0/1.0) as eluents. At the end, the solvent was distilled under reduced pressure and the residue was solubilized with ethyl acetate (10 mL); then a saturated solution of NaCl was added (5.0 mL). After the extraction with ethyl acetate (3 × 10 mL), the combined organic phases were washed with NaCl s.s. (10 mL), dried over Na_2_SO_4_, and filtered. The solvent was distilled under reduced pressure and the residue was purified by a silica gel chromatographic column using mixtures of dichloromethane and methanol (9.8:0.2, 9.5:0.5 or 9.0/1.0) as eluents. All compounds were characterized by NMR analysis. Tyrosyl and homovanillyl alcohol were obtained in yields ranging from 90 to 98%, and hydroxytyrosyl esters from 60 to 68%, as detailed in Table 1.

*4-Hydroxyphenethyl acetate (Tyrosyl acetate)***4**. Colorless oil. NMR spectra are in accordance with those reported in the literature [19,32,35].

*4-Hydroxyphenethyl butanoate (Tyrosyl butyrate)***5**. Colorless oil. NMR spectra are in accordance with those reported in the literature [32].

*4-Hydroxyphenethyl hexanoate (Tyrosyl hexanoate)***6**. Colorless oil. NMR spectra are in accordance with those reported in the literature [19,33].

*4-Hydroxyphenethyl octanoate (Tyrosyl caprylate)***7**. Yellow oil. NMR spectra are in accordance with those reported in the literature [33,35].

*4-Hydroxyphenethyl decanoate (Tyrosyl capriate)***8**. Yellow oil. NMR spectra are in accordance with those reported in the literature [33,35].

*4-Hydroxyphenethyl decanoate (Tyrosyl laurate)***9**. Yellow oil. NMR spectra are in accordance with those reported in the literature [32,35].

*4-Hydroxyphenethyl tetradecanoate (Tyrosyl myristate)***10**. Yellow oil. NMR spectra are in accordance with those reported in the literature [32,35].

*4-Hydroxyphenethyl palmitate (Tyrosyl palmitate)***11**. Colorless oil. NMR spectra are in accordance with those reported in the literature [19,32,35].

*4-Hydroxyphenethyl stearate (Tyrosol stearate)***12**. Colorless oil. NMR spectra are in accordance with those reported in the literature e [32,35].

*4-Hydroxyphenethyl oleate (Tyrosol oleate)***13**. Yellow oil. NMR spectra are in accordance with those reported in the literature e [32,35].

*4-Hydroxyphenethyl linoleate (Tyrosol linoleate)***14**. Yellow oil. NMR spectra are in accordance with those reported in the literature e [19,32,35].

*4-Hydroxy-3-methoxyphenethyl acetate (Homovanillyl acetate)***15**. Colorless oil. NMR spectra are in accordance with those reported in the literature [12,19].

*4-Hydroxy-3-methoxyphenethyl butanoate (Homovanillyl butyrate)***16**. Colorless oil. NMR spectra are in accordance with those reported in the literature [12].

*4-Hydroxy-3-methoxyphenethyl hexanoate (Homovanillyl hexanoate)***17**. Yellow oil. NMR spectra are in accordance with those reported in the literature [19].

*4-Hydroxy-3-methoxyphenethyl octanoate (Homovanillyl caprylate)***18**. Yellow oil. ^1^H-NMR (400 MHz, CDCl_3_) δ: 6.88–6.86 (1H, m, Ph-H), 6.74–6.72 (2H, m, Ph-H), 5.57 (1H, bs, OH), 4.27 (2H, t, J = 6.0 Hz, OCH_2_), 3.90 (3H, s, OCH_3_), 2.88 (2H, t, J = 6.0 Hz, Ph-CH_2_), 2.31 (2H, t, J = 8.0 Hz, COCH_2_), 1.60 (2H, m, CH_2_), 1.28 (8H, m, 4CH_2_), 0.90 (3H, m, CH_3_). ^13^C-NMR (100 MHz, CDCl_3_) δ: 173.4, 145.9, 143.8, 129.2, 121.1, 113.8, 110.9, 64.5, 55.4, 34.3, 33.9, 31.1, 28.6, 28.5, 24.5, 22.1, 13.5.

*4-Hydroxy-3-methoxyphenethyl decanoate (Homovanillyl capriate)***19**. Yellow oil. NMR spectra are in accordance with those reported in the literature [12].

*4-Hydroxy-3-methoxyphenethyl dodecanoate (Homovanillyl laurate)***20**. Yellow oil. ^1^H-NMR (400 MHz, CDCl_3_) δ: 6.97–6.95 (1H, m, Ph-H), 6.87–6.72 (2H, m, Ph-H), 5.40 (1H, bs, OH), 4.27 (2H, t, J = 8.0 Hz, OCH_2_), 3.89 (3H, s, OCH_3_), 2.88 (2H, t, J = 8.0 Hz, Ph-CH_2_), 2.31 (2H, t, J = 8.0 Hz, COCH_2_), 1.64–1.60 (2H, m, CH_2_), 1.28 (16H, m, 8CH_2_), 0.92–0.89 (3H, m, CH_3_). ^13^C-NMR (100 MHz, CDCl_3_) δ: 173.4, 145.9, 143.8, 129.1, 121.1, 113.9, 110.9, 64.5, 55.4, 34.3, 33.9, 31.4, 29.1, 29.0, 28.9, 28.8, 28.7, 28.5, 24.4, 22.1, 13.6.

*4-Hydroxy-3-methoxyphenethyl tetradecanoate (Homovanillyl myristate)***21**. Yellow oil. ^1^H-NMR (400 MHz, CDCl3) δ: 6.87–6.85 (1H, m, Ph-H), 6.74–6.72 (2H, m, Ph-H), 5.60 (1H, bs, OH), 4.27 (2H, t, J = 8.0 Hz, OCH_2_), 3.89 (3H, s, OCH_3_), 2.88 (2H, t, J = 8.0 Hz, Ph-CH_2_), 2.31 (2H, t, J = 8.0 Hz, COCH_2_), 1.64–1.60 (2H, m, CH_2_), 1.28 (20H, m, 10CH_2_), 0.92–0.89 (3H, m, CH_3_). ^13^C-NMR (100 MHz, CDCl3) δ: 173.4, 145.9, 143.8, 129.1, 121.1, 113.9, 110.9, 64.5, 55.4, 34.3, 33.9, 31.4, 29.1, 29.0, 28.9, 28.8, 28.7, 28.6, 28.5, 28.4, 24.5, 22.2, 13.6.

*4-Hydroxy-3-methoxyphenethyl palmitate (Homovanillyl palmitate)***22**. Colorless oil. NMR spectra are in accordance with those reported in the literature [19,30].

*4-Hydroxy-3-methoxyphenethyl stearate (Homovanillyl stearate)***23**. Yellow oil. NMR spectra are in accordance with those reported in the literature [12].

*4-Hydroxy-3-methoxyphenethyl oleate (Homovanillyl oleate)***24**. Yellow oil. NMR spectra are in accordance with those reported in the literature [19].

*4-Hydroxy-3-methoxyphenethyl linoleate (Homovanillyl linoleate)***25**. Yellow oil. NMR spectra are in accordance with those reported in the literature [19].

*3,4-Dihydroxyphenethyl acetate (Hydroxytyrosyl acetate)***26**. Yellow oil. NMR spectra are in accordance with those reported in the literature [12,19,46].

*3,4-Dihydroxyphenethyl butanoate (Hydroxytyrosyl butyrate)***27**. Colorless oil. NMR spectra are in accordance with those reported in the literature [4,12,44].

*3,4-Dihydroxyphenethyl hexanoate (Hydroxytyrosyl hexanoate)***28**. Yellow oil. NMR spectra are in accordance with those reported in the literature [19].

*3,4-Dihydroxyphenethyl octanoate (Hydroxytyrosyl caprylate)***29**. Colorless oil. NMR spectra are in accordance with those reported in the literature [34].

*3,4-Dihydroxyphenethyl decanoate (Hydroxytyrosyl capriate)***30**. Yellow oil. NMR spectra are in accordance with those reported in the literature [12,44].

*3,4-Dihydroxyphenethyl dodecanoate (Hydroxytyrosyl laurate)***31**. Yellow oil. NMR spectra are in accordance with those reported in the literature [31].

*3,4-Dihydroxyphenethyl tetradecanoate (Hydroxytyrosyl myristate)***32**. Yellow oil. NMR spectra are in accordance with those reported in the literature [30].

*3,4-Dihydroxyphenethyl palmitate (Hydroxytyrosyl palmitate)***33**. Yellow oil. NMR spectra are in accordance with the literature [19,31,44].

*3,4-Dihydroxyphenethyl stearate (Hydroxytyrosyl stearate)***34**. Yellow oil. NMR spectra are in accordance with those reported in the literature [12,31,44].

*3,4-Dihydroxyphenethyl oleate (Hydroxytyrosyl oleate)***35**. Colorless oil. NMR spectra are in accordance with those reported in the literature [19,31,44].

*3,4-Dihydroxyphenethyl linoleate (Hydroxytyrosyl linoleate)***36**. Colorless oil. NMR spectra are in accordance with those reported in the literature [19,31,44].

### 2.4. Esterification of Hydroxytyrosol Present in the Extracts: General Procedure

A total of 25 mg (0.16 mmol) of hydroxytyrosol-enriched extracts were dissolved in dimethyl carbonate (5.0 mL), and 0.19 mmol (40–200 μL) of the appropriate acyl chloride was introduced. The reaction was kept at 25°C under magnetic stirring for 24 h; then, dimethyl carbonate was distillated under reduced pressure by using a rotary evaporator (Laborota 4000, Heidolph, Munich, Germany). The mixture was solubilized with ethyl acetate and washed with a saturated solution of NaCl; then, the combined organic phases were dried over Na_2_SO_4_. After filtration, the solution was recovered, and the solvent was evaporated under reduced pressure. The content of hydroxytyrosol and the corresponding alkyl ester present in each sample was determined by HPLC–DAD analysis at λ = 280 nm. The yields of hydroxytyrosyl esters range from 60 to 66%.

## 3. Results and Discussion

**Scheme 1:**
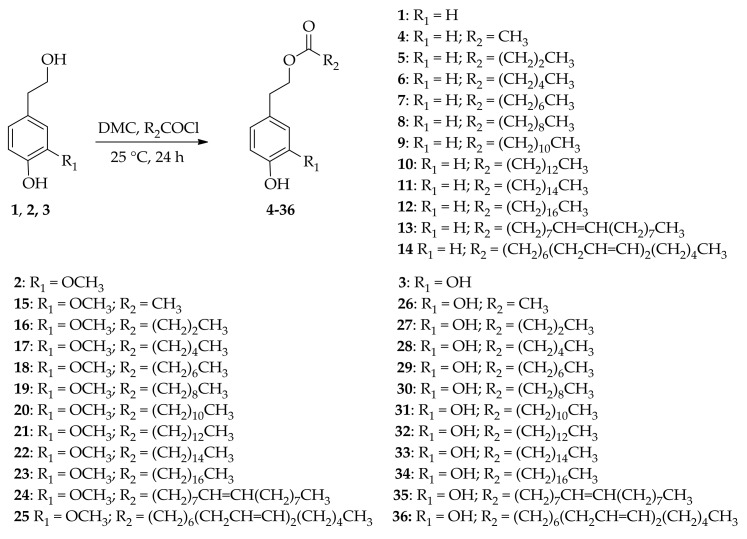
Esterification reactions of phenolic compounds **1**, **2** and **3**.

Tyrosol **1**, homovanillyl alcohol **2** or hydroxytyrosol **3** (Scheme 1) was solubilized in dimethyl carbonate (DMC), an eco-friendly solvent [47], and then a little excess of the appropriate acyl chloride (1.2 equiv.) was added. The reactions were stirred at room temperature for 24 h. After the work-up and column chromatographic purification, the corresponding esters were isolated in good yields (Table 1). The experimental results confirmed that the esterification reactions proceeded chemoselectively on the alcoholic group due the higher nucleophilicity compared to the phenolic moiety, emphasized by DMC, as already observed by us [19,47]. Even if hydroxytyrosol esters were isolated in lower yields compared to the enzymatic procedures reported in the literature [30,31,32,33,34,35], the simplicity of the operations and low cost of the reagents makes the described procedure attractive.

Most of the isolated esters exhibit a strong antioxidant activity in lipid media as oils and emulsions [33,35,41]. Tyrosol caprylate **7**, capriate **8**, and laurate **9** show remarkable antimicrobial activity against *Leishmania major*, *Leishmania infantum, Staphylococcus aureus*, *Staphylococcus xylosus*, *Bacillus cereus* and *Brevibacterium flavum* [35]. Hydroxytyrosol acetate **26**, found in olive oil [46], exhibits antioxidant activity in oil and emulsions [48]; hydroxytyrosol oleate **35**, recently found in olive oil by-products [49], is effective as an anti-inflammatory agent. Both derivatives **26** and **35** show antiproliferative activity on human cervical cells (HeLa) [50]. Hydroxytyrosol butanoate **27**, decanoate **30**, palmitate **33**, stearate **34**, oleate **35** and linoleate **36** are promising therapeutic agents for topical use in consideration to their cutaneous permeability [44].

Finally, hydroxytyrosol-enriched extracts were esterified under the same experimental conditions using the C2–C18 acyl chlorides. These samples were obtained by a selective extraction of *Olea europaea* by-products using a sustainable process based on membrane technologies [46]. The extracts contain 60.53 ± 0.41 mg/g of hydroxytyrosol (6.0 % w/w) on a total polyphenols content of 98.14 ± 2.43 mg/g [3,4]. After each esterification reaction, the hydroxytyrosyl ester found in the mixture was characterized and quantified by HPLC–DAD analysis. According to already observed using pure hydroxtytyrosol, the yields of the esterification reactions range from 60 to 66%.

Recently, we evaluated the antiproliferative activity of lipophilic fractions containing hydroxytyrosyl butanoate, octanoate and oleate on the human colon cancer cell line HCT8-β8, a model of colorectal cancer [4]. The experimental data has shown that all fractions exhibited antiproliferative activity. The relevant effect of hydroxytyrosol oleate was related to the high lipophilicity and bioavailability of the compound for the presence of the unsaturated C18 chain [4].

## 4. Conclusions

Lipophilic tyrosyl, homovanillyl and hydroxytyrosyl esters were prepared using a green and mild procedure. The products were obtained in good yields, solubilizing **1**, **2** or **3** in dimethyl carbonate, an eco-friendly solvent, and adding a little excess of the appropriate C2–C18 acyl chloride. The procedure was then applied to hydroxytyrosol-enriched extracts obtained by olive oil by-products to afford a panel of lipophilic fractions containing hydroxytyrosyl esters of different chain lengths to use for applications where the solubility in lipid media is required.

## Figures and Tables

**Table 1:** Esterification reactions of tyrosol **1**, homovanillyl alcohol **2** and hydroxytyrosol **3** (yields calculated after chromatographic purification).

*Substrate*	*Product*	*Yield (%)*	*Substrate*	*Product*	*Yield (%)*	*Substrate*	*Product*	*Yield (%)*
**1**	**4**	94	**2**	**15**	92	**3**	**26**	66
**1**	**5**	98	**2**	**16**	92	**3**	**27**	62
**1**	**6**	96	**2**	**17**	95	**3**	**28**	68
**1**	**7**	98	**2**	**18**	94	**3**	**29**	62
**1**	**8**	96	**2**	**19**	92	**3**	**30**	62
**1**	**9**	96	**2**	**20**	98	**3**	**31**	64
**1**	**10**	97	**2**	**21**	95	**3**	**32**	60
**1**	**11**	95	**2**	**22**	98	**3**	**33**	62
**1**	**12**	94	**2**	**23**	92	**3**	**34**	60
**1**	**13**	96	**2**	**24**	90	**3**	**35**	64
**1**	**14**	90	**2**	**25**	90	**3**	**36**	60

## References

[1] Bendini A., Cerretani L., Carrasco-Pancorbo A., Gomez-Caravaca A.M., Segura-Carretero A., Fernandez-Gutierrez A., Lercker G. (2007). Phenolic molecules in virgin olive oils: A survey of their sensory properties, health effects, antioxidant activiy and analytical methods. An overview of the last decade. Molecules.

[2] Della Greca M., Previtera L., Temussi F., Zarrelli A. (2004). Low-molecular weight components of olive oil mill wastewaters. Phytochem. Anal..

[3] Romani A., Pinelli P., Ieri F., Bernini R. (2016). Sustainability, innovation and green chemistry in the production and valorization of phenolic extracts from Olea europaea L.. Sustainability.

[4] Bernini R., Carastro I., Palmini G., Tanini A., Zonefrati R., Pinelli P., Brandi M.L., Romani A. (2017). Lipophilization of hydroxytyrosol-enriched fractions from *Olea europaea* L. by-products and evaluation of the in vitro effects on a model of colorectal cancer cells. J. Agric. Food Chem..

[5] Stahel W.R. (2016). The circular economy. Nature.

[6] Serino A., Salazar G. (2019). Protective role of polyphenols against vascular inflammation, aging and cardiovascular disease. Nutrients.

[7] Borzì A.M., Biondi A., Basile F., Luca S., Vicari E.S.D., Vacante M. (2018). Olive oil effects on colorectal cancer. Nutrients.

[8] In-Gyu Je I.-G., Kim D.-S., Kim S.-W., Lee S., Lee H.-S., Park E.K., Khang D., Kim S.-H. (2015). Tyrosol suppresses allergic inflammation by inhibiting the activation of phosphoinositide 3-kinase in mast cells. PLoS ONE.

[9] Saluccia S., Burattini S., Battistelli M., Buontempo F., Canonico B., Martelli A.M., Papa S., Falcieri E. (2015). Tyrosol prevents apoptosis in irradiated keratinocytes. J. Dermatol. Sci..

[10] Barontini M., Bernini R., Carastro R., Gentili P., Romani A. (2014). Synthesis and DPPH radical scavenging activity of novel compounds obtained from tyrosol and cinnamic acid derivatives. New J. Chem..

[11] Bernini R., Crisante F., D’Acunzo F., Gentili P., Ussia E. (2016). Oxidative cleavage of 1-aryl-isochroman derivatives by the Trametes villosa laccase/1-hydroxybenzotroazole system. New J. Chem..

[12] Grasso S., Siracusa L., Spatafora C., Renis M., Tringali C. (2007). Hydroxytyrosol lipophilic analogues: enzymatic synthesis, radical scavenging activity and DNA oxidative damage protection. Bioorg. Chem..

[13] Serreli G., Deiana M. (2018). Biological relevance of extra virgin olive oil polyphenols metabolites. Antioxidants.

[14] Goupy P., Dufour C., Loonis M., Dangles O. (2003). Quantitative kinetic analysis of hydrogen transfer reactions from dietary polyphenols to the DPPH radical. J. Agric. Food Chem..

[15] Bernini R., Merendino N., Romani A., Velotti F. (2013). Naturally occurring hydroxytyrosol: Synthesis and anticancer potential. Curr. Med. Chem..

[16] Bernini R., Gilardini Montani M.S., Merendino N., Romani A. (2015). Velotti, F. Hydroxytyrosol-derived compounds: a basis for the creation of new pharmacological agents for cancer prevention and therapy. J. Med. Chem..

[17] Fuccelli R., Fabiani R., Rosignoli P. (2018). Hydroxytyrosol exerts anti-inflammatory and anti-oxidant activities in a mouse model of systemic inflammation. Molecules.

[18] de Pablos R.M., Espinosa O., Ornedo-Hortega R., Cano M., Arguelles S. (2019). Hydroxytyrosol protects from aging process via AMPK and autophagy; a review of its effects on cancer, metabolic syndrome, osteoporosis, immune-mediated and neurodegenerative diseases. Pharmacol. Res..

[19] Bernini R., Mincione E., Barontini M., Crisante F. (2008). Convenient synthesis of hydroxytyrosol and its lipophilic derivatives from tyrosol or homovanillyl alcohol. J. Agric. Food Chem..

[20] Bernini R., Mincione E., Crisante F., Barontini M., Fabrizi G. (2009). A novel use of the recyclable polymer-supported IBX: an efficient chemoselective and regioselective oxidation of phenolic compounds. The case of hydroxytyrosol derivatives. Tetrahedron Lett..

[21] Bernini R., Cacchi S., Fabrizi G., Filisti E. (2008). 2-Arylhydroxytyrosol derivatives via Suzuki-Miyaura cross-coupling. Org. Lett..

[22] Bernini R., Crisante F., Merendino N., Molinari R., Soldatelli M.C., Velotti F. (2011). Synthesis of a novel ester of hydroxytyrosol and alpha-lipoic acid exhibiting an antiproliferative effect on human colon cancer HT-29 cells. Eur. J. Med. Chem..

[23] Bernini R., Crisante F., Fabrizi G., Gentili P. (2012). Convenient synthesis of 1-aryl-dihydroxyisochromans exhibiting antioxidant activity. Curr. Org. Chem..

[24] Fortunati E., Luzi F., Dugo L., Fanali C., Tripodo G., Santi L., Kenny J.M., Torre L., Bernini R. (2016). Effect of hydroxytyrosol methyl carbonate on the thermal, migration and antioxidant properties of PVA based films for active food packaging. Polym. Int..

[25] Fortunati E., Luzi F., Dugo L., Fanali C., Tripodo G., Santi L., Kenny J.M., Torre L., Bernini R. (2017). Hydroxytyrosol as active ingredient in poly(vinyl alcohol) films for food packaging applications. J. Renew. Mat..

[26] Luzi F., Fortunati E., Di Michele A., Pannucci E., Botticella E., Santi L., Kenny J.M., Torre L., Bernini R. (2018). Nanostructured starch combined with hydroxytyrosol in poly(vinyl) alcohol based ternary films as active packaging system. Carbohyd. Polym..

[27] Bovicelli P., Bernini R., Antonioletti R., Mincione E. (2002). Selective halogenation of flavanones. Tetrahedron Lett..

[28] Bernini R., Pasqualetti M., Provenzano G., Tempesta S. (2015). Ecofriendly synthesis of halogenated flavonoids and evaluation of their antifungal activity. New J. Chem..

[29] Bernini R., Barontini M., Cis V., Carastro I., Tofani D., Chiodo R.A., Lupattelli P., Incerpi S. (2018). Synthesis and evaluation of the antioxidant activity of lipophilic phenethyl trifluoroacetate esters by in vitro ABTS, DPPH and in cell-culture DCF assays. Molecules.

[30] Torres de Pinedo A., Penalver P., Rondon D., Morales J.C. (2005). Efficient lipase-catalyzed synthesis of new lipid antioxidants based on a catechol structure. Tetrahedron.

[31] Trujillo N., Mateos R., Collantes de Teran L., Espartero J.L., Cert R., Jover M., Alcudia F., Bautista J., Cert A., Parrado J. (2006). Lipophilic hydroxytyrosyl esters. Antioxidant activity in lipid matrices and biological systems. J. Agric. Food Chem..

[32] Mateos R., Trujillo M., Pereira-Caro G., Madrona A., Cert A., Espartero J. (2008). New lipophilic tyrosyl esters. Comparative antioxidant evaluation with hydroxytyrosyl esters. J. Agric. Food Chem..

[33] Lucas R., Comelles F., Alcántara D., Maldonado O.S., Curcuroze M., Parra J.L., Morales J.C. (2010). Surface-active properties of lipophilic antioxidants tyrosol and hydroxytyrosol fatty acid esters: a potential explanation for the nonlinear hypothesis of the antioxidant activity in oil-in-water emulsions. J. Agric. Food Chem..

[34] Tofani D., Balducci V., Gasperi T., Incerpi S., Gambacorta A. (2010). Fatty acid hydroxytyrosyl esters: structure/antioxidant activity relationship by ABTS and in cell-culture DCF assays. J. Agric. Food Chem..

[35] Aissa I., Sghair R.M., Bouaziz M., Laouini D., Sayadi S. (2012). Synthesis of lipophilic tyrosyl esters derivatives and assessment of their antimicrobial and antileishmanial activities. Lip. Health Dis..

[36] Bernini R., Crisante F., Barontini M., Tofani D., Balducci V., Gambacorta A. (2012). Synthesis and structure/antioxidant activity relationship of novel catecholic antioxidant structural analogues to hydroxytyrosol and its lipophilic esters. J. Agric. Food Chem..

[37] Pande G., Akoh C. (2016). Enzymatic synthesis of tyrosol‑based phenolipids: characterization and effect of alkyl chain unsaturation on the antioxidant activities in bulk oil and oil‑in‑water emulsion. J. Am. Oil. Chem. Soc..

[38] Sun Y., Zhou D., Shahidi F. (2018). Antioxidant properties of tyrosol and hydroxytyrosol saturated fatty acid. Food Chem..

[39] Zhou D.Y., Sun Y.X., Shahidi F. (2017). Preparation and antioxidant activity of tyrosoland hydroxytyrosol esters. J. Funct. Foods.

[40] Madrona A., Pereira-Caro G., Mateos R., Rodrıguez G., Trujillo M., Fernandez-Bolanos J., Espartero J.L. (2009). Synthesis of hydroxytyrosyl alkyl ethers from olive oil waste waters. Molecules.

[41] Shahidi F., Zhong Y. (2011). Revisiting the polar paradox theory: A critical overview. J. Agric. Food Chem..

[42] Akanbi T.O., Barrow C.J. (2018). Lipase-produced hydroxytyrosyl eicosapentaenoate is an excellent antioxidant for the stabilization of omega-3 bulk oils, emulsions and microcapsules. Molecules.

[43] Yin F.W., Hu X.P., Zhou D.Y., Ma X.C., Tian X.G., Huo X.K., Rakariyatham F., Shahidid F., Zhua B.-W. (2018). Evaluation of the stability of tyrosol esters during in vitro gastrointestinal digestion. Food Funct..

[44] Procopio A., Celia C., Nardi M., Oliverio M., Paolino D., Sindona G. (2011). Lipophilic hydroxytyrosol esters: fatty acid conjugates for potential topical administration. J. Nat. Prod..

[45] Pizzichini D., Russo C., Vitagliano M., Pizzichini M., Romani A., Ieri F., Pinelli P., Vignolini P. (2011). Process for producing concentrated and refined actives from tissues and byproducts of *Olea europaea* with membrane technologies. Eur. Pat..

[46] Brenes M., Garcia A., Garcia P., Rios J.J., Garrido A. (1999). Phenolic compounds in Spanish olive oils. J. Agric. Food Chem..

[47] Bernini R., Mincione E., Crisante F., Barontini M., Fabrizi G., Gentili P. (2007). Chemoselective and efficient carboxymethylation of the alcoholic chain of phenols by dimethyl carbonate (DMC). Tetrahedron Lett..

[48] Gordon M.H., Paiva-Martins F., Almeida M. (2001). Antioxidant activity of hydroxytyrosol acetate compared with other olive oil polyphenols. J. Agric. Food Chem..

[49] Plastina P., Benincasa C., Perri E., Fazio A., Augimeri G., Poland M., Witkamp R., Meijerink J. (2019). Indentification of hydroxytyrosyl oleate, a derivative of hydroxytyrosol with anti-inflammatory properties, in olive oil by-products. Food Chem..

[50] Bouallagui Z., Bouaziz M., Lassoued S., Engasser J.M., Ghoul M., Sayadi S. (2011). Hydroxytyrosol acyl esters: biosynthesis and activities. Appl. Biochem. Biotech..

